# Concentration-Dependent Anticancer Effects of Melittin-Functionalized Nanomaterials

**DOI:** 10.3390/ma19061134

**Published:** 2026-03-14

**Authors:** Alex Câmpian, Adrian Florea

**Affiliations:** Department of Cell and Molecular Biology, Faculty of Medicine, “Iuliu Hațieganu” University of Medicine and Pharmacy, 6 Louis Pasteur St., 400349 Cluj-Napoca, Romania; alexcampian@yahoo.com

**Keywords:** melittin, cancer, nanomaterials, dose–response, necrosis, apoptosis

## Abstract

Melittin-functionalized nanoparticles have emerged as a strategy to harness the potent anticancer activity of melittin while mitigating its narrow therapeutic window. Across diverse nanocarrier platforms, biological outcomes are highly dependent on the effective melittin concentration presented to tumour cells. This review systematically examines concentration-dependent anticancer effects of melittin-functionalized nanoparticles, focusing on quantitative dose–response metrics such as IC_50_ values, shifts in cytotoxic potency relative to free melittin, and concentration-linked safety margins. Along with some aspects concerning the molecular mechanisms of melittin, this review synthesizes evidence from preclinical studies to analyze how nanoparticle functionalization reshapes the concentration–effect relationship governing anticancer efficacy. This review concluded that there are three concentration regimes that govern the molecular outcome in tumours treated with melittin and melittin-functionalised nanomaterials. Collectively, the data demonstrate that nanoparticle association typically attenuates melittin’s intrinsic lytic potency, requiring higher nominal concentrations to achieve cytotoxicity, while simultaneously enabling tumour-selective re-potentiation through targeting, activation, or intracellular release mechanisms. These concentration-dependent phenomena define the translational limits and opportunities of melittin-based nanomedicines.

## 1. Introduction

Cancer remains one of the leading causes of morbidity and mortality worldwide and represents a major global health challenge. According to the latest global estimates from the International Agency for Research on Cancer, approximately 20 million new cancer cases and 9.96 million cancer-related deaths were recorded worldwide in 2022 [1]. Despite significant advances in chemotherapy, radiotherapy, immunotherapy, and targeted treatments, therapeutic resistance and systemic toxicity continue to limit clinical success. Tumour cells frequently develop resistance to conventional chemotherapeutic agents, leading to disease progression and recurrence. In this context, increasing attention has been directed toward bioactive natural compounds with alternative mechanisms of action, particularly those that exert direct membrane-disruptive effects and are less susceptible to classical resistance pathways. Among them, one of the oldest studied is melittin (Mel), the major molecule of honeybee venom, both in terms of composition—accounting for up to 50–55% of the venom dry weight [2,3]—and effects (reviewed in [4]).

Melittin attracted interest due to its potent biological activity against a wide range of cell types. Its ability to interact directly with lipid membranes enables the rapid disruption of membrane integrity, making Mel highly effective as a cytolytic, antimicrobial, and anticancer agent [5,6]. Beyond non-specific membrane lysis, Mel has also been shown to interfere with intracellular processes, including mitochondrial function and stress signalling pathways, contributing to its broad antitumour potential [2,7]. These properties position Mel as an unusually powerful therapeutic candidate, particularly in oncology, where resistance to conventional drugs remains a major challenge.

Despite this promise, the clinical translation of Mel has been severely limited by its lack of selectivity and narrow therapeutic window. It was found that Mel alone has limited cellular effects at low concentrations due to the preponderant localisation of its molecules in the plasma membrane. At increasing concentrations, free Mel readily disrupts the membranes of both malignant and healthy cells, leading to pronounced haemolysis and systemic toxicity when administered in vivo [2,8]. In addition, rapid degradation and uncontrolled biodistribution further complicate its therapeutic use. As a result, direct administration of Mel is incompatible with safe systemic therapy, necessitating strategies that can restrain its cytolytic activity while preserving anticancer efficacy. Nanomaterial-based functionalization has emerged as a leading approach to overcome these limitations. By incorporating Mel into nanoparticles or conjugating it to nanocarriers, researchers aim to shield the peptide from persistent membrane interactions, improve stability in biological environments, and enhance delivery to diseased tissues [8,9,10]. Such functionalization has been shown to reduce haemolysis, alter pharmacokinetics, and improve apparent tumour selectivity, thereby revitalizing interest in Mel as a viable therapeutic payload. Therefore, various nanocarriers were tested, aiming at increasing the cytoplasmic Mel concentration through their internalization by pinocytosis. Some of these platforms designed to transport Mel inside a wide range of cancer cells were presented in the outstanding reviews of Wang et al. 2022 [11] and Rizkallah et al. 2025 [12]. Thus, on one hand, Mel can be intracellularly delivered following its incorporation into poly-lactic acid–co-glycolic acid nanoparticles (MCF-7 human carcinoma breast cancer cells [13]), in different types of more or less complex liposomes (PANC1 pancreatic cancer cells [14]; MCF7 and SKBr3 human breast carcinoma cells [15]; multiple hepatic carcinoma cell lines: Bel-7402, BMMC-7721, HepG2, LM-3, and Hepa 1–6 cells [16]; B16F10 melanoma cells [17]; and A549 adenocarcinomic human alveolar basal epithelial cells [9]), into niosomes (4T1 and SKBR3 breast cells [18]) or their derivative bilosomes (PANC1 pancreatic cancer cells [19]); into other lipid nanoparticles (B16F10 melanoma cells [20]), MCF-7 and MDA-MB-231 breast cancer cells [21] (K7M2 osteoblast cells and osteosarcoma-bearing mice [22]); or into aluminum hydroxide oxide nanoscaffolds (4T1 cells in subcutaneous breast tumour models [23]). On the other hand, Mel was attached to complex lipodisks (A-431 epidermoid carcinoma cells [24] and U87 glioma cells in vitro and intracranial glioma models [25]) or to diverse solid nanostructures such as perfluorocarbon nanoparticles (C32 human melanoma cells [8]), chitosan nanocarriers (MDA-MB-231 cells [26]), carbon nanoparticles (MCF-7 and MDA-MB-231 cells [27]), nanodiamonds (MFC-7 cells [28]), iron oxide nanoparticles (MFC-7 cells [29]), gold nanoparticles (MFC-7 cells [30]), calcium carbonate nanoparticles (MCF-7 cells and C26 carcinoma cells [31]), silica nanoparticles (SKOV3 ovarian cancer cells [32]), MnO2 nanoparticles (B16, B16–F10, and B16-OVA melanoma cells, MC38 and MC38-OVA colon adenocarcinoma cells, and MB49 urothelial carcinoma cells [33]), etc.

However, while nanomaterial design has received extensive attention, the biological consequences of Mel delivery are often interpreted primarily in terms of carrier properties rather than the fundamental behaviour of the peptide itself.

A unifying feature across studies of free and nanoparticle-associated Mel is that Mel concentration is a central organizing variable. Independent of carrier composition, small changes in the amount of Mel delivered to cells can shift the balance between growth inhibition, programmed cell death, and non-specific membrane destruction [5,6,34]. Nanoparticle functionalization does not eliminate this concentration dependence; rather, it reshapes the spatial and temporal profile of Mel exposure. Consequently, differences in efficacy, toxicity, and selectivity reported for Mel-functionalized nanomaterials (Mel-NMs) often reflect differences in effective concentration rather than fundamentally distinct mechanisms of action.

This review has as its main scope and rationale an original in-depth analysis of the concentration-focused anticancer activity of Mel. This activity is intrinsically concentration-governed, with subtle changes in dose producing qualitatively distinct biological outcomes, ranging from apoptosis induction to non-specific membrane lysis. When Mel is functionalized onto nanoparticles, this concentration dependence is not eliminated but instead redistributed across multiple biological interfaces, including circulation, tumour accumulation, cellular uptake, and intracellular release.

Most existing reviews emphasize carrier design or mechanistic pathways while treating concentration as a secondary experimental parameter. However, across anticancer studies of Mel-NMs, reported half-maximal inhibitory concentration (IC_50_) values vary by more than an order of magnitude, even for similar tumour models, based on experimental conditions. This variability reflects not inconsistency but rather the central role of effective Mel concentration in determining biological outcome.

Accordingly, this review is structured mainly around quantitative concentration–response relationships, with emphasis on (1) changes in IC_50_ relative to free Mel; (2) concentration thresholds for apoptosis versus necrosis; and (3) dose ranges that preserve anticancer efficacy while reducing off-target toxicity.

This review adopts a concentration-centered framework to organize the literature but does not assume that concentration alone determines biological outcome in all melittin-functionalized systems. Rather, concentration is interpreted together with other modifying variables, including nanoparticle uptake, intracellular trafficking, organelle localization, release kinetics, exposure time, and tumour cell phenotype. The aim is therefore not to reduce all mechanistic diversity to a single parameter but to evaluate whether concentration provides a useful common axis for comparing otherwise heterogeneous studies.

For the main work, a structured literature search was conducted across PubMed, Web of Science, and Scopus up to 2025 using combinations of the following terms: *melittin*, *nanoparticle*, *nanocarrier*, *cancer*, *IC_50_*, *dose–response*, and *cytotoxicity*. Studies were included if they met all of the following criteria: (1) Mel was functionalized, encapsulated, or surface-associated with a nanoparticle system; (2) anticancer activity was assessed quantitatively using concentration-dependent assays; and (3) IC_50_ values or equivalent dose–response metrics were reported or extractable. To enable cross-study comparison, reported concentrations were normalized to micromolar (µM) Mel equivalents whenever possible.

## 2. Melittin: Synthesis and Molecular Mechanisms of Action

The honeybees of genus *Apis* (with 11 among the 17,533 species of bees classified by Michener [35]) synthesize complex venoms consisting of organic (peptides, proteins, amino acids, organic acids, amines, carbohydrates, and phospholipids) and inorganic (acids and salts) substances [2]. Mel is encoded in the bees’ cells by a single-copy gene [36]. As a secretion product, it is synthesized in the rough endoplasmic reticulum of cells forming the main venom gland [37] as a 70-amino-acid precursor, with a molecular weight of 7585 Da, and with the following sequence of amino acids [38]:

MKFLVNVALV^10^ FMVVYISYIY^20^ AAPEPEPAPE^30^ PEAEADAEAD^40^ PEAGIGAVLK^50^ VLTTGLPALI^60^ SWIKRKRQQG^70^

**Figure 1:**
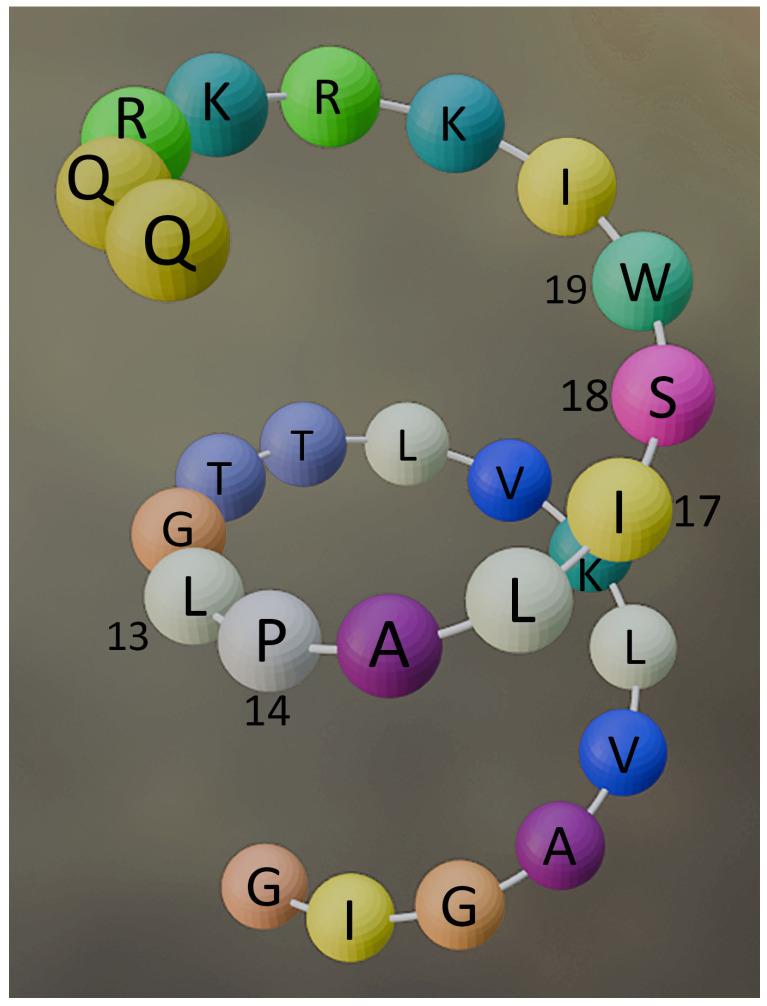
Representation of a melittin monomer.

The precursor is next processed by proteolytic cleavage to generate the mature form: the signal peptide of 21 amino acids (1–21), a smaller propeptide of 22 amino acids (22–43), and the last amino acids (70) are stepwise removed [38]. Zhou et al. perfected a chromatographic method for the precise quantification of Mel in lyophilized bee venom [39]. Following its purification, it was found that the secreted Mel is a small cationic peptide of 26 amino acids: GIGAVLKVLT^10^ TGLPALISWI^20^ KRKRQQ^26^ (Figure 1), with the molecular weight of 2846.53 Da [40], organized as a tetramer [2,41,42]. It has the chemical formula C_131_H_229_N_39_O_31_ [40]. In the aqueous environment of the venom sac, Mel is an α-helix (achieved by the trans conformation of the molecule at L^13^-P^14^ [43]) amphiphilic molecule, hydrophobic in the N-terminal region (20 amino acids), and strongly basic and hydrophilic in the region of the first 6 amino acids at the C-terminal end [2,44].

Mel modulates membrane water permeability, affecting all cells indiscriminately, following honeybee venom envenomation. When present in high doses, it triggers extensive haemolysis (being firstly described as a “direct haemolysin” [2], with severe renal complications [45,46,47,48], triggering the death of patients stung by high numbers of bees [47]. The unspecific ability of Mel to induce necrosis [49] results from its property to interact with membranes. This interaction depends on the chemical composition of the membrane and on the Mel concentration.

Mel enters in high amounts the hydrophobic regions of membranes consisting prevalently of electrically neutral phospholipids (phosphatidylcholines and sphingomyelins)—such as plasma membranes of cells, being responsible for pronounced effects [50]—and increasing the membrane permeability to water (measured with ^1^H-NMR method [51]). Also, the membrane surface charge [52] and the cholesterol content modulate the affinity of membranes for Mel, decreasing its insertion rate [53,54], thus limiting the cytolytic effects of Mel.

Mel triggers different types of changes in membranes when present at different concentrations. Dawson et al. showed that at relative low concentrations of Mel monomers in solution, the molecules partially enter the membrane with their N-terminal regions (the hydrophobic part) [55]. After an initial perpendicular binding of Mel at the membrane surface, the molecule monomers arrange with their helical axes [56] parallel to the membrane, and the Mel hydrophilic side comes in contact with the hydrophobic chains of the lipids. In a very short time, the helical packing of Mel is accelerated, and the monomers associate into tetramers [57]. This partial aggregation of the monomers (with their hydrophilic sides facing each other) is also essential to avoid their contact with the surrounding hydrophobic regions of phospholipids. If the Mel concentration is low, and according to certain physical (temperature, pH) or chemical (types of phospholipids) conditions, then the Mel monomers insert parallelly into the lipid bilayer, in the region of glycerol [58,59]. The three-dimensional structure of the crystalline form of Mel does not resist after the insertion into membranes [59]. The tertiary structure is developed under the conditions in which the energetic requirements for the atoms’ redistribution in the peptide chain are reduced by the hydrogen bonds [56]. Mel monomers perturb the orientation of lipid molecules extremely quickly (500 picoseconds), with different magnitudes of changes in the two monolayers [60]. By insertion into a biological membrane, Mel directly disturbs the mobility of lipids and reduces their lateral movements [61]. After insertion into the external monolayer, Mel associates in dimers or tetramers [59], with α-helixes involving 24 amino acids [62], the arrangement of lipids in the membrane is more deranged, and membrane disruption accentuates. The increasing concentration of Mel in membranes results in the aggregation of the intrinsic proteins [63], and eventually the disintegration of the bilayer, following removal of lipid–protein assemblies surrounded by Mel molecules [59]. From a more concentrated solution, large amounts of Mel arrive in the membrane, where it adopts a tetrameric helix conformation, oriented perpendicularly to the lipid bilayer, with the tendency to cross it [41,59]. However, its complete transmembrane configuration is never achieved since the Mel molecule has a single polar region. The Mel tetramers produce changes in the membrane phospholipid arrangement and organize small pores. In the structure of these pores, the Mel α-helixes are separated by hydrophilic heads of phospholipids, and the resulting pores are not organized as classic channels with central hydrophilic regions [64,65]. They are called toroidal pores because of the way that some lipids curve in, resulting in an ideal shape for Mel to interact with. These pores firstly allow the passage of water molecules, the residues I^17^-S^18^-W^19^ close to the C-termini of Mel chains being implicated in the process [66]. Using fluorescent markers entrapped in artificial phospholipid vesicles and released under the action of different concentrations of Mel, ref. [67] estimated an average pore diameter produced by Mel of around 2.5–3 nm. The same diameter of the Mel pores was found in the RBC membrane [68]. Ladokhin and White showed that a decisive factor in defining the mechanism of action of Mel in membranes is represented by their composition [67]. Thus, in the liposomes consisting of positively charged phospholipids, Mel produces pores according to the later-described mechanism. Apart from these mechanisms, in the anionic phospholipid vesicles, Mel does not form pores, but following its parallel insertion acts as a detergent, leading to the complete solubilization of the vesicles.

On the other hand, Mel was studied for its ability to induce apoptosis in different type of cells, including malignant cells, with different groups of researchers reporting several mechanisms involved in the process. Thus, Zhang and Chen reported the in vitro apoptosis of human non-small cell lung cancer cells (cell line A549) as a response to 2 µg/mL Mel, as well as a significant in vivo dose-dependent reduction in tumours in mice after 24 days of administration of Mel (1 and 10 mg/kg). At the molecular level, these authors showed that Mel inhibited the epithelial growth factor-induced migration and invasion of cancer cells by inhibiting the expression of vascular endothelial growth factor and hypoxia-inducible factor-1α with the increasing concentration of Mel [69]. Furthermore, Gao et al. demonstrated with in vitro and in vivo xenograft models that Mel triggered (at 2 μg/mL) the apoptosis of non-small cell lung cancer cells (NCI-H441 cell line) by activating caspase-2 (via inhibition of miR-183 expression, elevation of Bax, and reduced Bcl-2 protein expression), resulting in decreased invasion and migration abilities of malignant cells. The authors also achieved the suppression of non-small cell lung cancer tumour growth by the subcutaneous injection of Mel (at 5 mg/kg) [70].

Nikodijević et al. tested Mel on human colorectal adenocarcinoma cells (HT-29 cell line) and reported concentrations of 1 and 5 µg/mL Mel inducing apoptosis after 24 h, increasing the expression of the Fas receptor, and by upregulating the gene expression and activity of caspase 8, respectively [71].

In general, the methods used to evaluate Mel in the context of apoptosis induction mechanisms also, to a large extent, detect cellular necrosis, and the markers assessed differ between these processes. To improve accuracy, we therefore aim to further analyze the effects of Mel-NP.

## 3. Concentration Framework: Three Regimes of Activity

A unifying feature across studies of free Mel and Mel-NMs is the strong dependence of biological outcome on effective Mel concentration. Regardless of delivery platform, cell type, or experimental system, Mel-induced cytotoxicity follows reproducible concentration-dependent patterns that reflect the peptide’s intrinsic membranolytic properties [5,6]. Nanoparticle functionalization alters the spatial and temporal distribution of Mel exposure but does not eliminate the fundamental concentration thresholds that govern cellular response [8,72].

On this basis, the biological effects of Mel can be organized into three distinct concentration regimes, each associated with characteristic cellular outcomes and therapeutic implications. This framework provides a conceptual lens through which diverse experimental observations can be reconciled and serves as the organizing principle for the analyses presented in subsequent sections.

**Figure 2:**
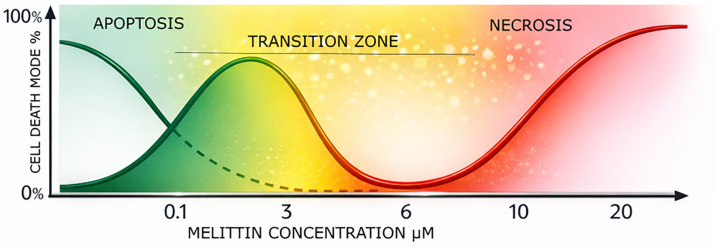
Conceptual schematic of the concentration-dependent transition between predominantly apoptotic and predominantly necrotic cellular responses to melittin exposure.

To avoid conflating mechanistic diversity with dose effects, anticancer outcomes were analyzed across three concentration-defined functional zones. The first one, the sub-lytic concentration regime, is typically present at Mel concentrations below 2 µM. It is characterized by growth inhibition, mitochondrial dysfunction, and apoptosis. These changes are frequently observed only after nanoparticle-mediated intracellular accumulation. The second one is the intermediate cytotoxic regime, with doses of ~2–6 µM effective Mel. It is associated with classical IC_50_ values in nanoparticle systems. There is a balance between membrane perturbation and programmed cell death. The final concentration regime is the supra-lytic, which arouses at above 6 µM effective Mel. In this case, non-specific membrane disruption dominates. Also, the main issue is the reduced tumour selectivity despite enhanced cytotoxicity (Figure 2). This framework is applied consistently throughout the review to interpret how nanoparticle functionalization shifts Mel activity along the concentration axis.

### 3.1. Sub-Lytic Regime

**Figure 3:**
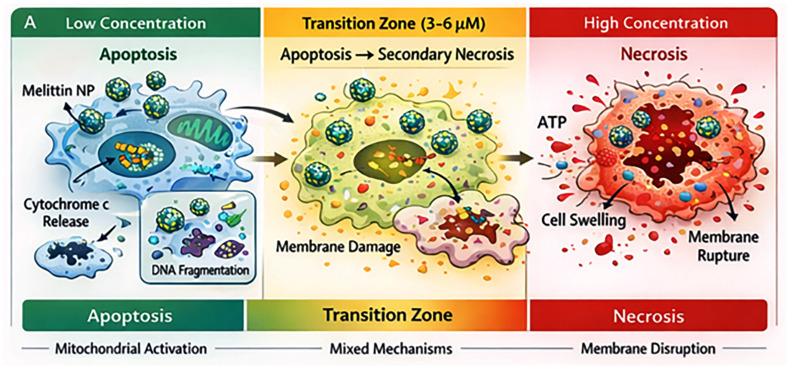
Conceptual summary of the dominant cellular effects associated with low, intermediate, and high extracellular melittin-equivalent concentrations in melittin-functionalized nanoparticle systems.

At low effective concentrations, typically in the range of approximately ≤2 µM, Mel exposure does not result in immediate plasma membrane disruption. Instead, cells exhibit growth inhibition, metabolic stress, and early signs of programmed cell death (Figure 3). In this sub-lytic regime, overt membrane lysis is minimal, and cell death—when it occurs—is predominantly apoptotic in nature [8].

This concentration range is rarely accessible with free Mel due to its rapid interaction with the plasma membrane [6]. However, nanoparticle-mediated delivery can delay membrane exposure and promote intracellular accumulation, allowing sub-lytic concentrations to exert biological effects before catastrophic membrane damage occurs [7,8]. The sub-lytic regime therefore represents the lowest concentration range in which anticancer activity becomes detectable while systemic toxicity remains limited.

### 3.2. Intermediate Cytotoxic Regime

As effective Mel concentrations increase into an intermediate range, typically ~2–6 µM, cytotoxicity becomes more pronounced and reproducible across tumour models. This regime corresponds to the optimal therapeutic window observed for many Mel-NM systems [8,72,73]. Within this window, sufficient intracellular exposure is achieved to induce robust tumour cell killing, while non-specific membrane disruption and off-target toxicity remain partially constrained.

Biologically, this regime is characterized by a balance between controlled membrane perturbation and the activation of regulated cell death pathways (Figure 3). Nanoparticle systems are most effective in this concentration range, where gradual release kinetics and heterogeneous intracellular distribution can extend the window between initial cytotoxic signalling and irreversible membrane failure [12,72]. Consequently, many reports of enhanced selectivity or reduced hemolysis associated with nanoparticle delivery can be understood as the stabilization of Mel exposure within this intermediate regime rather than as fundamental alterations of Mel activity.

### 3.3. Supra-Lytic Regime

At higher effective concentrations, generally ≥8 µM, Mel’s intrinsic membranolytic activity dominates biological outcome (Figure 3). In this supra-lytic regime, rapid plasma membrane disruption, uncontrolled ion flux, and loss of cellular homeostasis result in non-specific necrotic cell death [2,5]. Tumour selectivity is markedly reduced, as membrane interactions overwhelm the modulatory effects of nanoparticle delivery and affect both malignant and non-malignant cells [8].

Importantly, this regime defines a hard upper boundary for therapeutic application. Across studies, dose–response curves for Mel-NMs converge toward those of free Mel at high concentrations, underscoring that nanoparticle systems delay but do not abolish concentration-dependent toxicity [8,72].

### 3.4. Implications of the Concentration Framework

Together, these three regimes delineate a concentration-governed continuum of biological responses to Mel exposure. Nanoparticle functionalization primarily reshapes the probability of occupying a given regime by altering delivery kinetics, intracellular localization, and release profiles, rather than by redefining the underlying thresholds themselves [72,74]. This framework provides a foundation for interpreting apparent shifts in potency, selectivity, and safety reported across the literature and motivates a focus on concentration control as the central design challenge for Mel-based nanotherapeutics.

In the following sections, this concentration framework is applied to analyze reported IC_50_ shifts, dose–response curve morphology, mechanistic transitions between apoptosis and necrosis, and the design strategies employed to modulate effective Mel concentration in vitro and in vivo.

## 4. IC_50_ Shifts & Tumour Variability

### 4.1. Comparative Analysis of IC_50_ Shifts Relative to Free Melittin

When directly compared, free Mel consistently demonstrates IC_50_ values between 0.5 and 4 µM across most cancer cell lines (Table 1).

In contrast, Mel-NMs exhibit IC_50_ values ranging from ~1 µM to >30 µM, depending on carrier design, functionalization chemistry, and targeting strategy. This apparent reduction in potency has occasionally been misinterpreted as diminished efficacy. However, concentration-dependent analysis reveals that higher nominal IC_50_ values correspond to fundamentally different biological interactions, including (1) reduced membrane insertion per unit concentration; (2) increased intracellular routing prior to cytotoxic action; and (3) delayed but sustained exposure within tumour cells. Importantly, studies employing receptor-targeted nanoparticles consistently demonstrated partial restoration of low-micromolar IC_50_ values, suggesting that effective intracellular concentration, rather than applied dose, is the dominant determinant of anticancer activity [81].

### 4.2. Concentration-Dependent Shifts in IC_50_ Following Nanoparticle Functionalization

One consistent observation across anticancer studies is that nanoparticle association often increases the apparent IC_50_ of Mel relative to free peptide, consistent with partial shielding of membranolytic activity. In perfluorocarbon lipid nanoparticles with surface-associated Mel, IC_50_ values shifted from ~0.9–2.2 µM (free Mel) to ~28.9–32.3 µM (non-targeted nanoparticles) in melanoma and endothelial cell proliferation assays, while αvβ3 targeting reduced IC_50_ to ~6.8–8.4 µM [8,82].

In redox-responsive polymeric nanocomplexes, cytotoxicity depends strongly on formulation and targeting chemistry, with targeted constructs showing higher potency than non-targeted complexes in 24 h viability assays, consistent with intracellular activation/release effects [83].

Across studies, these shifts underscore that nanocarrier design primarily reshapes Mel’s concentration–effect relationship (and selectivity/toxicity window) rather than increasing intrinsic peptide potency.

### 4.3. Variability of IC_50_ Values Across Tumour Cell Types

A consistent observation across studies of Mel-NMs is that cytotoxicity becomes strongly dependent on cellular uptake mechanisms and intracellular processing rather than on the intrinsic membranolytic activity of the peptide. In perfluorocarbon nanoparticle systems bearing surface-associated Mel, IC_50_ values shifted from 0.9 to 2.2 µM for free Mel to 28–32 µM for non-targeted nanoparticles in C32 human melanoma cells and 2F2B murine endothelial cells, while αvβ3-targeted nanoparticles reduced IC_50_ to 6.8–8.4 µM, demonstrating uptake-dependent restoration of potency [8]. These findings indicate that nanoparticle-mediated delivery amplifies cell-specific concentration effects rather than overriding intrinsic peptide cytotoxicity. Consistent with this, Cheng et al. showed that redox-sensitive polymeric Mel nanocomplexes exhibited enhanced selective cytotoxicity toward cancer cells relative to non-triggered constructs, reflecting intracellular release triggered by elevated glutathione levels rather than direct membrane disruption [83]. Similar variability in dose–response behaviour has been observed across cell lines and formulations, underscoring that cellular context and intracellular trafficking strongly modulate the effective concentration–response relationship of Mel delivered via nanocarriers.

### 4.4. Tumour-Specific Accumulation and the Local Concentration Challenge

Achieving the therapeutic concentration window in vivo is fundamentally limited by transport barriers and the heterogeneity of the tumour microenvironment. While nanoparticles utilize the enhanced permeability and retention effect to improve delivery, recent analyses suggest that the actual accumulation of administered doses often remains below required thresholds [84,85].

To reach the 2–6 µM intratumoural concentration required for efficacy, Mel-NPs must overcome biological barriers such as high interstitial fluid pressure and dense extracellular matrix [86,87,88]. Effective nanomedicine design must balance systemic stability—often through PEGylation or liposomal encapsulation [89,90,91,92]. Evidence suggests that targeted formulations consistently achieve intratumoural concentrations in the 2–6 µM range, corresponding to the optimal window of selective cytotoxicity identified in vitro.

### 4.5. Concentration-Driven Heterogeneity Within Tumour Cell Populations

Single-cell analyses have revealed substantial intra-population heterogeneity in re-sponse to Mel-NMs, particularly at intermediate concentrations near the IC_50_. At doses between 2 and 6 µM, some cells exhibited early apoptotic markers while others remained viable, resulting in broadened dose–response curves [8,12]. This heterogeneity diminished at higher concentrations, where uniform necrosis predominated. Such findings suggest that nanoparticle-mediated Mel delivery introduces stochastic variability in intracellular concentration, influenced by differences in nanoparticle uptake and intracellular traffick-ing (Table 2).

From a therapeutic standpoint, this variability underscores the importance of dose optimization, as intermediate concentration regimes may allow partial tumour survival and potential resistance development.

## 5. Dose–Response Morphology and Time Dependence

### 5.1. Time–Concentration Interdependence in Anticancer Activity

In contrast to free Mel, whose cytotoxic effects often manifest rapidly at high concentrations, Mel-NMs frequently demonstrate time-dependent potentiation at lower concentrations. This temporal dimension complicates direct IC_50_ comparison but provides important insight into how nanoparticle systems modulate effective concentration over time.

Melittin-loaded niosomes, for example, displayed IC_50_ values of approximately 143 µg/mL at 24 h, decreasing to ~75 µg/mL at 72 h in MCF-7 cells, indicating progressive intracellular accumulation or delayed release of Mel [18,93]. Free Mel, by contrast, exhibited minimal time-dependent enhancement, suggesting rapid membrane interaction rather than sustained intracellular exposure [94].

Similar trends were observed in lipid–polymer hybrid nanoparticles, where low micromolar concentrations produced negligible cytotoxicity at 12 h but resulted in >50% viability loss by 48–72 h [9]. These findings support a model in which nanoparticle functionalization converts Mel from an acute lytic agent into a concentration-accumulating cytotoxin, with IC_50_ values that decrease over time.

### 5.2. Concentration Thresholds for Selective Cytotoxicity

A critical advantage of Mel-NMs is the emergence of selective cytotoxic concentration windows, wherein tumour cells are preferentially affected relative to non-malignant controls. Across multiple studies, this selectivity was consistently observed within a narrow intermediate concentration range, typically between 2 and 6 µM.

For instance, polymeric nanoparticles presenting surface-bound Mel induced apoptosis in glioblastoma cells at concentrations of 2.5 µM while maintaining viability in primary astrocytes at the same dose [12]. Above 10 µM, however, selectivity was lost, with both malignant and non-malignant cells exhibiting extensive membrane disruption.

This concentration-dependent selectivity contrasts sharply with free Mel, which rarely exhibits differential toxicity across cell types due to its immediate and non-specific membrane activity at sub micromolar concentrations [7]. Thus, nanoparticle functionalization does not broaden the therapeutic window by lowering required doses, but rather by creating a concentration regime in which tumour-selective mechanisms dominate.

### 5.3. Dose–Response Curve Morphology and Slope Differences

**Figure 4:**
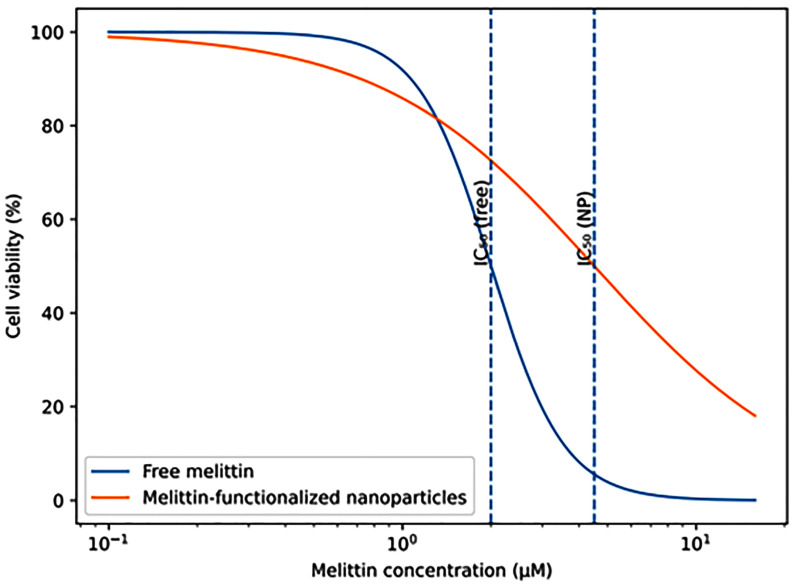
Illustrative comparison of dose–response curve morphology for free melittin versus melittin-functionalized nanoparticle systems.

Beyond IC_50_ values, the shape of dose–response curves provides additional insight into concentration-dependent behaviour. Free Mel typically produces steep, sigmoidal curves, reflecting cooperative membrane disruption and rapid transition from viability to lysis within a narrow concentration range. In contrast, Mel-NMs frequently exhibit shallower dose–response slopes (Figure 4), with gradual reductions in viability across a broader concentration range [8,72]. This flattening suggests heterogeneous intracellular delivery and reinforces the concept that nanoparticle systems distribute Mel exposure over time and space, rather than delivering an immediate bolus. From a translational perspective, these altered dose–response characteristics may reduce the risk of catastrophic toxicity from minor dosing errors, even if higher nominal concentrations are required.

### 5.4. Time–Concentration Interactions and Apparent Potency

Concentration-dependent effects of Mel-NMs cannot be interpreted independently of exposure time. Several studies demonstrated that sub-IC_50_ concentrations (1–2 µM) induce significant cytotoxicity when exposure exceeds 48–72 h, whereas higher concentrations (>5 µM) achieve comparable effects within 12–24 h [9,18,72,83]. This inverse relationship between concentration and required exposure time suggests that Mel-mediated membrane damage accumulates in a quasi-linear fashion until a critical threshold is reached. Nanoparticle formulations slow this accumulation by modulating release kinetics, effectively trading peak concentration for prolonged exposure. However, prolonged exposure at concentrations approaching haemolytic thresholds increases the risk of delayed systemic toxicity in vivo [95,96]. Thus, optimal therapeutic strategies appear to involve moderate concentrations with controlled release, rather than prolonged low-level exposure or high-dose bolus administration.

## 6. Mechanistic Transitions: Apoptosis ↔ Necrosis

Mechanistic studies indicate that Mel-induced cell death mechanisms shift as a function of concentration. At lower concentrations (≤2 µM), nanoparticle-delivered Mel predominantly induces mitochondrial dysfunction, caspase activation, and apoptosis [12,97]. Intermediate concentrations (2–6 µM) are associated with mixed apoptotic and necrotic features, while higher concentrations (>8–10 µM) result in rapid membranolysis and necrosis, regardless of delivery system [98]. These shifts have important therapeutic implications. Apoptotic cell death is generally associated with reduced inflammatory signalling and improved tolerability, whereas necrotic death can exacerbate local inflammation and systemic toxicity. Nanoparticle systems that restrict effective Mel concentration to the apoptotic range may therefore offer qualitative as well as quantitative therapeutic advantages.

### 6.1. Mitochondria

Mitochondria have been identified as a critical intracellular target contributing to Mel-induced cytotoxicity. Multiple studies demonstrate that Mel can trigger the collapse of mitochondrial membrane potential, promote cytochrome C release, and activate the intrinsic apoptotic pathway in cancer cells [99,100,101]. Importantly, mitochondrial dysfunction has been observed at concentrations that induce apoptotic signalling without immediate overt plasma membrane lysis, indicating that mitochondrial perturbation can precede extensive membranolysis in certain cellular contexts [99,102,103]. Biophysical studies performed by Li et al. confirm that Mel interacts robustly with cardiolipin-containing model membranes, supporting a preferential destabilization mechanism at the mitochondrial level [102]. At higher extracellular concentrations, Mel’s well-characterized ability to insert into and permeabilize plasma membranes becomes dominant, resulting in rapid membrane disruption and mixed apoptotic–necrotic phenotypes [5,101]. Because free Mel rapidly associates with the plasma membrane, intracellular organelle targeting may be limited under systemic exposure conditions. In contrast, nanoparticle-mediated delivery systems have been shown to enhance intracellular accumulation and reduce non-specific membrane lysis, thereby increasing the likelihood of mitochondrial engagement as a cytotoxic mechanism [8]. These findings suggest that mitochondrial targeting represents a low-concentration cytotoxic mechanism that is largely inaccessible to free Mel due to its rapid interaction with the plasma membrane.

### 6.2. Lysosomes

In addition to mitochondrial targeting, lysosomal accumulation has been implicated in the concentration-dependent effects of Mel-NMs. Endocytosed nanoparticles frequently traffic to lysosomal compartments, where localized Mel concentrations may exceed cytosolic levels, even when extracellular doses are relatively low. Several studies reported lysosomal membrane permeabilization at low extracellular Mel concentrations, leading to cathepsin release and downstream apoptotic signalling [8,104,105]. Notably, this effect was strongly dose-dependent: sub-threshold concentrations (<2 µM) failed to induce lysosomal disruption, whereas supra-threshold concentrations (>8 µM) caused generalized membrane damage independent of lysosomal pathways. These observations support a two-stage concentration model, in which moderate extracellular doses generate high local intracellular concentrations within endolysosomal compartments, triggering apoptosis, while higher doses overwhelm compartmentalization and produce non-specific cytotoxicity.

### 6.3. Reactive Oxygen Species

Reactive oxygen species (ROS) generation represents another mechanism by which concentration-dependent effects are amplified in Mel nanoparticle systems. A dose-dependent increase in intracellular ROS following exposure to Mel-NMs, with significant ROS accumulation occurring at concentrations near the IC_50_. At 2–5 µM, ROS generation was sufficient to activate stress-responsive kinases and promote apoptotic signalling without inducing oxidative membrane damage. At higher concentrations (>8–10 µM), ROS levels exceeded cellular antioxidant capacity, contributing to lipid peroxidation and necrosis [102]. This further highlights how nanoparticle-mediated delivery enables concentration-dependent intracellular amplification mechanisms that are otherwise inaccessible.

### 6.4. Apoptosis–Necrosis Transition as a Function of Melittin Concentration

One of the most consequential concentration-dependent phenomena observed in Mel-NM systems is the qualitative shift in the mode of tumour cell death. Unlike free Mel, which predominantly induces rapid necrotic lysis at submicromolar concentrations, nanoparticle-associated Mel frequently induces programmed cell death at intermediate concentrations, with necrosis emerging only at higher exposure levels. Across multiple studies, apoptosis predominated at effective Mel concentrations between approximately 1 and 6 µM, as evidenced by caspase-3 activation, mitochondrial membrane depolarization, and DNA fragmentation [8,99,102]. Within this concentration window, plasma membrane integrity was largely preserved during early exposure, suggesting that nanoparticle-mediated delivery delays direct membrane disruption and allows intracellular signalling pathways to dominate. At concentrations exceeding 8–12 µM, a transition toward necrotic morphology was consistently reported, characterized by lactate dehydrogenase release, rapid ATP depletion, and loss of membrane integrity [8]. Importantly, this apoptotic–necrotic transition occurred at higher concentrations for nanoparticle-associated Mel than for free Mel, reinforcing the notion that nanoparticle functionalization shifts Mel’s cytotoxic action along the concentration axis.

### 6.5. Concentration-Dependent Apoptosis–Necrosis Transition Induced by Melittin-Functionalized Nanoparticles

The biological response to Mel-NMs is governed by a finely balanced interplay between local Mel concentration, exposure kinetics, and subcellular distribution. While nanocarrier systems profoundly alter biodistribution and circulation half-life, they do not negate the fundamental concentration-dependent membranolytic nature of Mel. Instead, nanoparticle functionalization reshapes the spatiotemporal concentration profile of Mel, thereby modulating the dominant mode of cell death. Although concentration provides a useful organizing axis for these transitions, the observed balance between apoptosis and necrosis in melittin-functionalized systems is also shaped by intracellular trafficking, endolysosomal processing, organelle exposure, release kinetics, and possible carrier-specific biological effects. Thus, the apoptotic tendency observed in nanoparticle systems should not be interpreted as a concentration effect alone.

### 6.6. Concentration as a Central Organizing Variable in Melittin Cytotoxicity

Melittin is an amphipathic, cationic peptide whose biological activity scales non-linearly with concentration. At increasing local concentrations, Mel transitions from transient membrane perturbation to stable pore formation and ultimately to catastrophic membrane disintegration [5,6]. This intrinsic behaviour underlies the sharp switch between apoptotic and necrotic cell death observed across multiple experimental systems.

Nanoparticle functionalization typically results in a rightward shift in the dose–response curve, with IC_50_ values increasing by 2-fold to over 20-fold compared to free Mel. This underscores that nanoparticle design primarily acts by reshaping Mel’s concentration–effect relationship rather than enhancing its intrinsic potency [8]. By shielding the peptide from immediate membrane contact, nanoparticles allow Mel to accumulate intracellularly prior to exerting full membranolytic activity [82,89].

### 6.7. Low-Concentration Regime: Mitochondrial-Driven Intrinsic Apoptosis

At low effective intracellular concentrations, Mel-NPs consistently induce classical intrinsic apoptosis across a range of cancer cell lines. Hallmark features include mitochondrial membrane depolarization, cytochrome c release, and caspase activation.

This apoptotic bias reflects the differential sensitivity of mitochondrial membranes, which are enriched in cardiolipin [106,107]. Nanoparticle-mediated delivery allows Mel to bypass the plasma membrane and preferentially engage these mitochondrial targets [8,99,102].

### 6.8. Intermediate Concentration Regime: Apoptosis–Necrosis Overlap and Secondary Necrosis

As effective concentrations rise into the intermediate range (~2–6 µM), a mechanistic transition zone emerges where apoptotic and necrotic features coexist. Cells often display partial caspase activation, declining ATP levels, and delayed plasma membrane permeability [69,72,108].

Time-resolved analyses reveal that many cells initially undergo apoptosis but subsequently progress to secondary necrosis due to bioenergetic exhaustion [34,97,108]. This intermediate concentration range often defines the upper limit of the therapeutic window for Mel-based nanotherapeutics.

### 6.9. High-Concentration Regime: Dominant Primary Necrosis

At high effective concentrations (≥8 µM), the modulatory influence of nanoparticle delivery is overwhelmed. Rapid plasma membrane rupture and necrotic morphology are observed within minutes of exposure [6,11]. This necrotic regime is associated with pronounced inflammatory responses and off-target tissue injury [34,109].

## 7. Modulators of Effective Concentration

### 7.1. Targeting Strategies as Modulators of Effective Melittin Concentration

While nanoparticle functionalization generally increases the nominal concentration required for cytotoxicity, tumour-targeting strategies consistently re-shift the concentration–response curve leftward, restoring anticancer efficacy at lower extracellular doses. This phenomenon reflects enhanced local and intracellular Mel concentration rather than changes in intrinsic peptide activity. In ligand-targeted perfluorocarbon nanoparticle systems, the incorporation of α_vβ_3-integrin-binding motifs reduced IC_50_ values from approximately 28–32 µM to 6–8 µM in melanoma models [8]. Importantly, this reduction occurred without a corresponding increase in haemolytic activity, indicating that targeting selectively increases tumour cell exposure without elevating systemic concentration. These findings underscore that targeting strategies primarily act by increasing the effective intracellular concentration achieved per unit dose, rather than by altering release kinetics alone.

### 7.2. Comparative Efficacy of Activation Strategies Across Concentration Regimes

When evaluated comparatively, targeting and activation strategies differ in how they reshape the concentration–response relationship: (1) targeting ligands reduce IC_50_ by increasing uptake efficiency; (2) protease activation introduces sharp concentration thresholds; and (3) pH- and redox-responsiveness amplify intracellular concentration while preserving low extracellular exposure. Across systems, maximal selectivity was consistently observed within an administered concentration range of 2–6 µM, beyond which control mechanisms became saturated [8,9,20,94,110]. These findings suggest that nanoparticle engineering cannot compensate for poor dose selection, and that concentration remains a key variable governing biological outcome (Table 3 and Table 4). Despite marked methodological heterogeneity, the studies summarized in Table 3 show a recurrent directional pattern rather than a single universal potency range. In general, nanoparticle association shifts apparent IC_50_ values rightward relative to free melittin, consistent with partial shielding of direct membranolytic activity, whereas active targeting or stimulus-responsive activation can partially restore potency in tumour-associated settings. Because these values were generated under different exposure times, assay platforms, and dose-reporting conventions, they are most informative for identifying formulation-dependent trends rather than establishing a strict potency hierarchy across studies.

### 7.3. Enzyme-Activated Nanoparticles and Concentration Threshold Control

Protease-activated Mel nanoparticles represent a distinct approach to concentration modulation, in which Mel activity is suppressed until enzymatic cleavage occurs within the tumour microenvironment. In these systems, effective Mel concentration is decoupled from administered dose, allowing high systemic exposure without immediate cytotoxicity. Matrix metalloproteinase-responsive nanoparticles have demonstrated minimal cytotoxicity at concentrations up to 20 µM in non-tumourigenic cells, while inducing apoptosis in tumour cells at local effective concentrations estimated to exceed 3–5 µM following enzymatic activation [112,113]. This sharp concentration threshold effect enables a binary response: inactive below the activation threshold, and potently cytotoxic once local concentration exceeds it. Such systems exhibit unusually steep dose–response curves post-activation, resembling free Mel behaviour but confined spatially to the tumour microenvironment. This spatial concentration control represents one of the most effective strategies for exploiting Mel’s anticancer potential while minimizing off-target toxicity.

### 7.4. pH-Responsive Release and Intracellular Concentration Amplification

Tumour acidity has been exploited to trigger Mel release from pH-sensitive nanoparticles, further refining concentration-dependent behaviour. In several lipid-based and polymeric systems, Mel release was minimal at physiological pH but accelerated dramatically below pH 6.5, corresponding to late endosomal and lysosomal environments [114,115,116].

In vitro studies demonstrated that extracellular doses of 2–4 µM Mel equivalents produced negligible cytotoxicity at neutral pH, yet induced >60% apoptosis under acidic conditions [11]. This suggests that intracellular pH gradients amplify local Mel concentration, effectively shifting the dose–response curve without increasing administered dose. Notably, supra-threshold extracellular concentrations (>10 µM) overwhelmed pH-dependent control mechanisms, resulting in non-specific toxicity regardless of pH. This finding highlights the importance of maintaining administered doses within the concentration range where stimuli-responsive modulation remains operative.

### 7.5. Redox-Triggered Release and Concentration Synchronization

Redox-responsive systems, typically exploiting elevated intracellular glutathione levels in tumour cells, have also been shown to fine-tune concentration-dependent effects. Disulfide-linked Mel nanocomplexes remain largely inactive extracellularly but release Mel upon intracellular reduction, producing synchronized intracellular concentration spikes. In colorectal cancer models, these systems achieved IC_50_ values of ~1–2 µM, despite minimal membrane interaction at equivalent extracellular concentrations [83]. This indicates that redox-triggered release can effectively compress Mel exposure into a short intracellular time window, mimicking high local concentration without systemic toxicity. At higher administered doses (>8 µM), however, premature reduction and partial release occurred extracellularly, eroding selectivity.

Thus, even in advanced activation systems, concentration control remains a significant determinant of therapeutic outcome (Table 5).

## 8. In Vivo Constraints and Transport Barriers

### In Vivo Translation: Concentration Constraints Beyond Cell Culture

In Vivo studies consistently report that effective plasma concentrations of Mel equivalents rarely exceed 1–3 µM, even when higher doses are administered [11]. This discrepancy reflects rapid clearance, protein binding, and distributional dilution, underscoring the importance of local concentration amplification at tumour sites. Tumour accumulation studies using labelled Mel nanoparticles have estimated intratumoural concentrations in the range of 2–6 µM, corresponding closely to in vitro IC_50_ values for targeted systems [20,110,115]. These findings support the notion that successful in vivo efficacy depends on achieving tumour-local concentrations within the narrow therapeutic window identified in vitro, rather than on high systemic exposure. Notably, formulations that produced transient plasma concentrations exceeding 5 µM were frequently associated with acute toxicity, even when haemolysis was minimized in vitro [18]. This highlights limitations of erythrocyte-based safety assays and suggests that vascular endothelium and immune cells may represent additional concentration-sensitive targets.

## 9. Failure Modes and Therapeutic Window Modelling

### 9.1. Failure Modes at High Concentrations

Several studies reported that even the most sophisticated targeting or activation systems failed to prevent toxicity when administered Mel concentrations exceeded 10–15 µM [11]. At these levels, non-specific membrane interactions dominated, and dose–response curves converged toward those observed for free Mel. This convergence emphasizes a fundamental limitation: nanoparticle functionalization modulates but does not eliminate Mel’s intrinsic concentration-dependent toxicity. Translational success therefore depends not on maximal dosing but on the precise alignment of dose, delivery, and activation thresholds.

### 9.2. Implications for Concentration Selection in Preclinical Models

Taken together, these data indicate that anticancer efficacy and mechanism are inseparable from concentration selection in Mel-NM systems. Concentrations optimized solely for maximal cytotoxicity may inadvertently favour necrosis and loss of selectivity, whereas concentrations tuned to exploit apoptotic and organelle-specific mechanisms may offer superior therapeutic indices. Preclinical studies that fail to report full dose–response relationships risk obscuring these distinctions, highlighting the need for standardized concentration reporting and mechanistic stratification.

## 10. Design Principles for Melittin Nanotherapeutics

### 10.1. Therapeutic Window Modelling in Melittin-Functionalized Nanoparticles

One of the most consistent findings across studies is that nanoparticle functionalization does not abolish Mel toxicity but reshapes the therapeutic window by separating efficacious and haemolytic concentration ranges. Modelling of dose–response curves indicates that the therapeutic window is primarily determined by the horizontal displacement between tumour cell IC_50_ and erythrocyte HD_50_ values. For free Mel, this displacement is minimal or absent, with IC_50_ and HD_50_ values often overlapping within 0.5–2 µM [5,8]. Nanoparticle formulations typically shift haemolytic thresholds rightward by one to two orders of magnitude, while anticancer IC_50_ values shift more modestly, resulting in a window of selective cytotoxicity spanning approximately 2–6 µM depending on formulation [8]. Importantly, studies employing quantitative pharmacodynamic modelling have shown that maximal therapeutic indices occur at intermediate concentrations rather than at maximal tolerated doses [116,117]. This observation challenges conventional oncologic dosing paradigms and emphasizes the need for precision concentration control rather than dose escalation in Mel-based nanotherapeutics.

Nanoparticle functionalization does not abolish melittin toxicity, but it can improve the apparent therapeutic window by separating haemolytic activity from anticancer potency. For free melittin, IC_50_ and HD_50_ values often lie in a similar low-micromolar range, indicating little safety margin. By contrast, nanoparticle loading, targeting, or conditional activation can shift haemolytic thresholds rightward more strongly than tumour cell IC_50_ values, thereby creating a wider interval of selective activity. Representative studies supporting this pattern are shown in Table 6. Because these measurements are highly assay-dependent, they should be interpreted as indicating directional improvement in therapeutic separation rather than a universal therapeutic index.

HD_50_ and haemolysis values are assay-dependent and vary with erythrocyte source, incubation time, and experimental conditions. Accordingly, the studies shown here are intended to illustrate representative directional changes in therapeutic separation rather than to define a universal therapeutic index.

### 10.2. Methodological Variability in Concentration Reporting

A significant limitation in the current literature is the lack of standardization in reporting Mel concentration. Studies variably report concentrations as (1) total Mel loaded; (2) free Mel equivalents; (3) nanoparticle mass-based dosing; and (4) estimated intracellular concentrations. This heterogeneity complicates cross-study comparisons and meta-analytic synthesis. Several authors have advocated for reporting both administered concentration and measured free Mel release, along with time-resolved concentration profiles [117,118]. Without such standardization, apparent differences in potency between formulations may reflect measurement artefacts rather than true biological effects. Future studies should prioritize quantitative concentration metrics to enable rational comparison and optimization.

### 10.3. Design Principles Derived from Concentration-Dependent Behaviour

Synthesizing data across systems reveals several robust design principles: (1) efficacy plateaus beyond ~6–8 µM, while toxicity increases sharply; (2) targeting strategies are most effective below 6 µM administered concentration; (3) activation-based systems require precise dose calibration to avoid premature release; and (4) nanoparticle complexity cannot compensate for supra-therapeutic concentrations. These principles suggest that the next generation of Mel nanotherapeutics should prioritize dose precision, pharmacokinetic predictability, and real-time concentration monitoring rather than further increases in delivery sophistication.

## 11. Conclusions

The biological activity of Mel-NMs is governed fundamentally by concentration-dependent effects, with nanoparticle design serving as a modulatory, rather than transformative, factor. Across diverse systems, a narrow concentration window consistently emerges in which anticancer efficacy is maximized and systemic toxicity minimized.

Based on the analytical framework provided in the review, the relationship between Mel concentration and cell death pathways can be visualized as a shift in the mode of action along a concentration axis.

As Mel concentration increases, the biological outcome transitions through three distinct phases:

- Sub-lytic Regime (≤2 µM): In this range, cell death is primarily characterized by growth inhibition, mitochondrial dysfunction, and apoptosis. This is frequently observed only after nanoparticle-mediated intracellular accumulation.
- Intermediate Cytotoxic Regime (~2–6 µM): This represents the “optimal therapeutic window” where nanoparticle systems demonstrate selective cytotoxicity. Here, a balance exists between membrane perturbation and apoptosis.
- Supra-lytic Regime (>6 µM): At these high levels, non-specific necrosis and membrane disruption dominate. Tumour selectivity is significantly reduced as non-specific membrane interactions overwhelm nanoparticle control mechanisms.

From a nanomedicine design perspective, these findings imply that optimal Mel delivery systems should be engineered not to maximize intracellular accumulation but to stabilize intracellular concentrations within the apoptotic window. Strategies that promote gradual release, heterogeneous intracellular distribution, or conditional activation below necrotic thresholds are therefore more likely to yield clinically viable formulations than approaches emphasizing maximal payload delivery.

From a translational standpoint, the concentration-dependent transition between apoptosis and necrosis has direct implications for dose selection, escalation strategies, and safety margins in preclinical development. Nanoparticle systems that flatten dose–response curves and widen the apoptotic window may offer improved robustness against interpatient variability and local concentration heterogeneity, reducing the risk of catastrophic toxicity arising from modest dosing deviations.

Ultimately, Mel-NMs succeed not by overcoming concentration-dependent toxicity, but by temporarily aligning Mel exposure with the narrow biological window in which its cytotoxic potential can be exploited without loss of selectivity.

## Figures and Tables

**Table 1:** Free Melittin IC_50_ across several tumour cell lines.

Tumour Cell Line(s)	IC_50_ (µM)	Exposure Time	Assay	Ref.
MCF-7 (breast)	3.6 µM	24 h	MTT	[75]
Hepa 1–6 (mouse liver)	2.24 µM	24 h	MTT	[75]
A549, NCI-H460, NCI-H1975 (lung)	1.5–3.0 µM (1.52, 1.79, 2.98 µM)	24 h	PrestoBlue viability	[76]
HCT116 (colon)	1.34 µM	24 h	MTT	[77]
A375 (malignant melanoma)	1.19 µM	24 h	MTT	[78]
SK-MEL-28 (melanoma)	1.08 µM	72 h	MTT	[79]
K562 (CML leukemia)	0.65 µM	24 h?	MTT	[80]

**Table 2:** Reported IC_50_ values of free vs. nanoparticle-functionalized melittin in cancer cell lines.

Cancer Model	Formulation	IC_50_ (µM)	Exposure Time	Key Notes	Ref.
C32 human melanoma	Free Mel	0.93 ± 0.26	3 h (MTT)	High cytotoxicity, strong haemolysis	[8]
C32 human melanoma	Perfluorocarbon NP (non-targeted)	32.3 ± 3.9	3 h	~35-fold rightward IC_50_ shift; reduced acute toxicity	[8]
C32 human melanoma	αvβ3-targeted perfluorocarbon NP	6.8 ± 2.1	3 h	Targeting partially restores potency	[8]
HepG2 hepatocellular carcinoma	Mel nano-liposome	~2–4 (time-dependent)	24–48 h	Reduced acute toxicity; apoptosis predominance	[16]
MCF-7 breast cancer	Mel-loaded niosomes	16.45	48 h	Reduced hemolysis; time-dependent cytotoxicity	[18]
Multiple tumour cell lines	Lipid-coated polymeric NP	2.5–6	24–72 h	Shielding reduces early lysis; delayed apoptosis	[9]
Cancer cell lines	Redox-sensitive polymeric nanocomplex	1	24 h	Glutathione-triggered intracellular release; reduced haemolysis	[83]

**Table 3:** Reported IC_50_ values of melittin-functionalized systems under heterogeneous experimental conditions.

Formulation/ Functionalization	Tumour Model	Assay	Exposure	Reported IC_50_	Normalized IC_50_ (µM Melittin Equation)	Ref.
Free melittin	B16F10 melanoma	MTT	12 h	0.75 ± 0.19 µM	0.75 ± 0.19 µM	[9]
Perfluorocarbon melittin nanoemulsion	B16F10 melanoma	MTT	12 h	5.1 ± 1.2 µM	5.1 ± 1.2 µM	[9]
Free melittin	C32 melanoma	MTT	3 h	~1–2 µM	~1–2 µM	[9]
αvβ3-targeted perfluorocarbon melittin nanoparticle	C32 melanoma	MTT	3 h	~6–8 µM	~6–8 µM	[9]
Non-targeted perfluorocarbon melittin nanoparticle	C32 melanoma	MTT	3 h	>25 µM	>25 µM	[9]
Native melittin	2F2B endothelial model	Cytotoxicity assay	~3 h	2.4 µM	2.4 µM	[111]
MMP-9-activatable melittin prodrug nanoparticle	2F2B endothelial model	Cytotoxicity assay	~3 h	>100 µM	>100 µM	[111]
MMP-9-cleaved activated melittin prodrug nanoparticle	2F2B endothelial model	Cytotoxicity assay	~3 h	8.1 µM	8.1 µM	[111]
Redox-sensitive melittin nanocomplex	HCT116 colorectal carcinoma	MTT	24 h	4.15 µM	4.15 µM	[83]
LBA-targeted redox-sensitive melittin nanocomplex	HCT116 colorectal carcinoma	MTT	24 h	1.0 µM	1.0 µM	[83]
Melittin-loaded niosomes	A549 NSCLC	Cytotoxicity assay	72 h	0.69 µg/mL	0.24 µM	[93]
Melittin-loaded niosomes	Calu-3 NSCLC	Cytotoxicity assay	72 h	1.02 µg/mL	0.36 µM	[93]
Melittin nano-liposomes	Bel-7402/BMMC-7721/HepG2/LM-3/Hepa1–6 hepatic carcinoma lines	Survival/apoptosis assays	not specified	1.44–2.1 µM	1.44–2.1 µM	[16]

**Table 4:** Concentration thresholds associated with biological outcomes. Concentration ranges represent approximate extracellular melittin equivalents derived from multiple studies.

Effective Melittin Concentration	Dominant Biological Effect	Notes
<1 µM	Minimal cytotoxicity	Often sub-therapeutic
1–2 µM	Apoptosis	Caspase activation, mitochondrial damage
2–6 µM	Mixed apoptosis/necrosis	Optimal therapeutic window
6–10 µM	Rapid necrosis	Loss of selectivity
>10 µM	Non-specific membrane lysis	High systemic toxicity

**Table 5:** Effect of nanoparticle design on concentration–response curve shifts.

Strategy	Shift in IC_50_	Shift in HD_50_	Net Effect
PEGylation	↑ (5–20×)	↑↑ (20–100×)	Safety gain
Active targeting	↓ (2–5×)	Neutral	Efficacy recovery
Enzyme activation	Binary threshold	↑↑	Spatial selectivity
pH/redox activation	↓ intracellular	Neutral	Intracellular amplification

↑—moderate increase; ↑↑—high increase; ↓—moderate decrease.

**Table 6:** Representative studies comparing haemolytic toxicity and anticancer potency in melittin-based systems.

System	HD_50_/Hemolysis	IC_50_/Anticancer Potency	Main Implication	Ref.
Free melittin	Hemolytic and cytotoxic ranges largely overlap	0.75 ± 0.19 µM in B16F10; ~1–2 µM in C32	High potency, but poor safety separation	[9]
Non-targeted melittin nanoparticle	Hemolysis reduced relative to free melittin	5.1 ± 1.2 µM in B16F10; >25 µM in C32	Nanoparticle loading reduces acute lytic activity but also shifts IC_50_ rightward	[9]
αvβ3-targeted melittin nanoparticle	Hemolysis still reduced relative to free melittin	~6–8 µM in C32	Targeting partially restores potency while preserving improved safety	[9]
Free melittin	HD_50_ = 1.9 µM	IC_50_ = 2.4 µM	Minimal separation between lysis and anticancer activity	[111]
MMP-9-activatable melittin prodrug NP (inactive state)	HD_50_ > 100 µM	IC_50_ > 100 µM	Strong suppression of off-target lytic activity	[111]
MMP-9-activated melittin prodrug NP	HD_50_ = 3.4 µM	IC_50_ = 8.1 µM	Activity is restored after activation, with partial safety separation maintained	[111]
Redox-sensitive melittin nanocomplex	Reduced hemolysis reported relative to free melittin	4.15 µM (non-targeted); 1.0 µM (targeted HCT116)	Triggered/targeted delivery can recover potency without reverting fully to free-melittin toxicity	[83]

## Data Availability

No new data were created or analyzed in this study. Data sharing is not applicable to this article.

## Abbreviations

The following abbreviations are used in this manuscript:

IC_50_	Half maximal inhibitory concentration
Mel	Melittin
Mel-NM	melittin-functionalized nanomaterials
